# Supplementary material for: Chromothripsis during telomere crisis is independent of NHEJ, and consistent with a replicative origin
Source: Genome Res. 2019 May;29(5):737–49. doi: 10.1101/gr.240705.118 (PMC6499312; doi:10.1101/gr.240705.118)
Supplement: Supplemental Material [file supp_gr.240705.118_Supplemental_file_1.zip › contigs/annotated_contigs/DB55/contig.2.DB55_length_369_mean_cov_7.70731707317.docx]

**DB55_length_369_mean_cov_7.70731707317**

```
CATCTCTGACGTCTGTTGCCCACTCCTCCCCATCCCCTGCCTCCTCCATTCATTCATTCAACACAGATTTACTGTGTCCTGTGACGCAG
 >chr7:6870385-6870707 - E=7e-180
GCACTGTGTTAGACAACAGGAACCAGAAGATGGCCCCAACAGAGTCCTGGCCTCAAGAAGTTCACTTTCCTTGATAGGAAGGAGGGCAT

GGTGGCCCGTGCCTGTAGGACCAGCTATATAGGAGGCAGAGGCAGGAGGATCGCTTGAGCCCAGGAGTTAGGGGCTGCAGTGAGCTATG

ACCATACCACTGCACTATAAACTGGGCAACAGAGCAAGACTGTCTCAAACCTA|TA|ACGTGGGCTGTGATCGCACACTTCCACTCCAG
                                                      >chrUn_gl000220:120777-120826 + E
CTTACGTGGGCTGCG=5e-18
```
